# Ligation of 2′, 3′‐cyclic phosphate RNAs for the identification of microRNA binding sites

**DOI:** 10.1002/1873-3468.13976

**Published:** 2020-11-16

**Authors:** Christian Berk, Yuluan Wang, Artur Laski, Stylianos Tsagkris, Jonathan Hall

**Affiliations:** ^1^ Institute of Pharmaceutical Sciences Department of Chemistry and Applied Biosciences ETH Zurich Switzerland; ^2^ Present address: Max Planck Institute of Immunobiology and Epigenetics Freiburg Germany

**Keywords:** 2′,3′‐cyclic phosphate, CLIP, ligation, microRNA, RtcB

## Abstract

Identifying the targetome of a microRNA is key for understanding its functions. Cross‐linking and immunoprecipitation (CLIP) methods capture native miRNA‐mRNA interactions in cells. Some of these methods yield small amounts of chimeric miRNA‐mRNA sequences *via* ligation of 5′‐phosphorylated RNAs produced during the protocol. Here, we introduce chemically synthesized microRNAs (miRNAs) bearing 2′‐, 3′‐cyclic phosphate groups, as part of a new CLIP method that does not require 5′‐phosphorylation for ligation. We show in a system that models miRNAs bound to their targets, that addition of recombinant bacterial ligase RtcB increases ligation efficiency, and that the transformation proceeds *via* a 3′‐phosphate intermediate. By optimizing the chemistry underlying ligation, we provide the basis for an improved method to identify miRNA targetomes.

## Graphical abstract

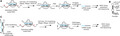

## Abbreviations

**ACN**, acetonitrile

**CLIP**, cross‐linking and immunoprecipitation

**CPG**, controlled pore glass

**DMT**, dimethoxytrityl

**miRISC**, miRNA‐induced silencing complex

**miRNAs**, microRNAs

**nt**, nucleotides

**THF**, tetrahydrofuran

**TBDMS**, tertButyldimethylsilyl

**Fig. 1:**
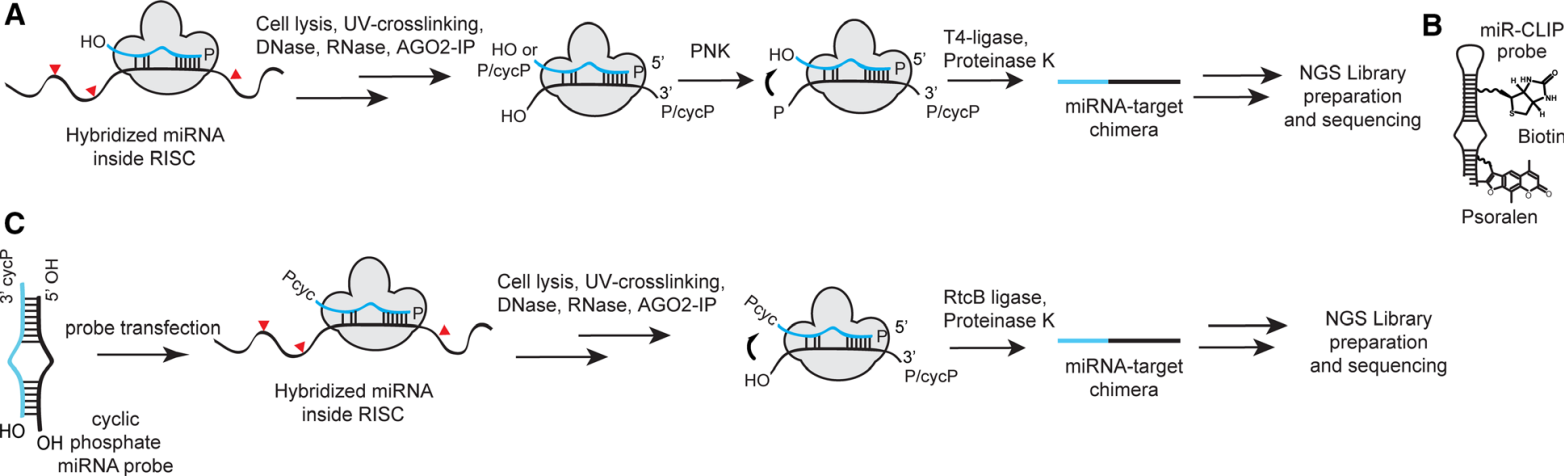
Cross‐linking strategies of miRNAs to their mRNA targets in CLIP protocols. (A) Ligation of miRNA‐mRNA target pairs in miRISC after controlled treatment of the target mRNA with RNase A or T1 during CLASH [15] or CLEAR‐CLIP [14] methods; chimeras are formed with low efficiency after 5′‐phosphorylation and T4‐ligase mediated end‐joining. RNA sequencing reveals the miRNA, its mRNA target, and the site of interaction. (B) MiR‐CLIP probes use psoralen to cross‐link to their target mRNAs. MiRNA‐mRNA target pairs are enriched by biotin–streptavidin enrichment [6]. (C) The proposed protocol (this study). MiRNA‐mRNA chimeras may form with high efficiency after RtcB‐mediated ligation of cyclic phosphate‐terminated miRNA probes to the 5′‐hydroxyl terminus of the target RNA. RNA sequencing reveals the miRNA, its mRNA target, and the site of interaction.

MicroRNAs (miRNAs) are small RNAs of about 20 nucleotides (nt) in length that bind to conserved sites in mRNAs and suppress gene expression. MiRNAs engage their targets in the miRNA‐induced silencing complex (miRISC) containing an argonaute protein. Typically, binding of the miRNA to an mRNA is governed by a stretch of about seven complementary base pairs at the 5′ end of the miRNA (seed region). However, non‐canonical interactions, for example, involving the central or 3′ terminal parts of miRNAs are increasingly reported [1, 2, 3, 4, 5, 6, 7]. In order to help determine the importance of noncanonical miRNA‐mRNA interactions to the regulation of gene expression, methods are needed that identify all of the mRNA targets of a miRNA (its targetome) in cells. A variety of cross‐linking and immunoprecipitation (CLIP) methods exist to capture native miRNA‐mRNA interactions in miRISC, which are identified by RNA sequencing [8, 9]. In native CLIP approaches (e.g., PAR‐CLIP [10, 11, 12, 13]), all of the miRNA‐mRNA interactions in the cell are captured indiscriminately and individual interactions are subsequently inferred using bioinformatics tools. However, native CLIP methods do not identify precisely the miRNA family member and the target sites implicated in cross‐linking events. In the CLEAR‐CLIP [14] and CLASH [15] variants of CLIP, ligated chimeras from some miRNA‐mRNA complexes are formed (Fig. 1A). Thus, the sequencing data identifies the miRNA, its associated mRNA, as well as the precise site of interaction. This complete picture of an interaction provides valuable insight into miRNA functions, but unfortunately, the efficiency of chimera formation is generally so low that many sites are represented by only single chimeric reads [16]. Recently, we described an alternative target‐capture approach (miR‐CLIP [6]) that uses chemically synthesized miRNAs that are site‐specifically labeled with psoralen and biotin groups (Fig. 1B). In cells, miR‐CLIP reagents are accepted into miRISC and function as native miRNAs. Upon mild irradiation, they cross‐link to their mRNA targets and after a work‐up involving enrichment steps, the mRNA targets of this single defined miRNA are revealed by RNA sequencing. Like the native CLIP methods, miR‐CLIP is also unable to identify the precise site of interaction between the miRNA and its target.

2′, 3′‐Cyclic phosphate‐terminated RNAs (RNA>p) are a distinct class of RNAs with their own properties [17, 18]. Cyclic phosphate species and their enzyme‐mediated ligation reactions are well known in the field of tRNA splicing [19, 20] and are postulated to have played roles in the ‘RNA world’ hypothesis [21, 22, 23, 24, 25]. Whereas in conventional ligation mechanisms, an activated 5′‐phosphate group of an RNA is attacked by a 3′‐hydroxyl group of a second ribonucleotide [26, 27, 28], RNA>p participate in an unusual mechanism of ligation that was first observed in HeLa cell lysates and involves the attack of a 5′‐hydroxyl RNA [19, 39]. In mammalian cells, it occurs preferentially with RNA>p, but also with 3′‐phosphate RNAs that are first cyclized by 3′‐phosphate cyclase [19, 37, 38, 39]. This reaction was assumed to be responsible for the chimera formation during CLIP‐type experiments which did not involve a dedicated ligation step [14, 40]. The tRNA ligase HSPC117 was presumed to be the enzyme responsible for this transformation [14, 19], however, neither overexpression nor depletion of HSPC117 in cells significantly changes the rate of chimera formation [14].

Recent studies on the bacterial tRNA ligase RtcB—an orthologue of HSPC117—provided evidence for a Mn^2+^‐ and GTP‐dependent ligation, which proceeds *via* prior hydrolysis of the cyclic phosphate [29, 30, 31, 32, 35, 36]. In this pathway, RtcB mediates transfer of GMP to the RNA 3′‐phosphate terminus, whereupon this RNA 3′‐ppG intermediate is then attacked by a ribonucleotide 5′‐hydroxyl group to form a 3′‐5′‐phosphodiester linkage. We describe here the first steps toward exploiting this mechanism as the basis of a new miR‐CLIP method that is intended to generate miRNA‐mRNA chimeras in high yields (Fig. 1C). We first designed and synthesized miRNAs bearing cyclic phosphate groups at their 3′‐ends (miRNA>p). We demonstrate that in cells these RNAs are accepted into miRISC and suppress their targets similarly to native miRNAs. We show that in a model system which mimics miRNA‐mRNA duplexes in miRISC, a miR‐34a probe (miR‐34a>p) is ligated to partially complementary RNAs by addition of recombinant RtcB and that ligations proceed *via* a 3′‐phosphate intermediate [29, 30, 31, 32, 35, 36]. In optimizing this chemistry, we provide the basis for testing a novel means to identify the targetome of a miRNA in cells.

## Material and methods

### Oligonucleotide synthesis

Chemicals for oligonucleotide synthesis were purchased from Sigma‐Aldrich (Steinheim, Germany), Fluorochem (Hadfield, UK), and TCI (Eschborn, Germany). 5′‐DMT‐2′‐*O*‐TBDMS phosphoramidites were obtained from Thermo Fisher Scientific (Waltham, MA, USA). Oligonucleotides were synthesized on a MM12 synthesizer from Bio Automation (BioAutomation Corp., Irving, TX, USA) using 50 nmol synthesis columns (BioAutomation) and 5 mg of either 1000 Å UnyLinker controlled pore glass (CPG) (ChemGenes, Wilmington, MA, USA) or 5′‐DMT‐2′, 3′‐cyclic phosphate adenosine (N‐Bz), uridine, cytidine (N‐Ac) or guanosine (N‐iBu) 1000 Å CPG (catalog no N‐8501‐XX; ChemGenes). Phosphoramidites were prepared as 0.08 m solutions in dry acetonitrile (ACN), the activator 5‐(Benzylthio)‐1H‐tetrazole (BTT; Biosolve BV, Valkenswaard, Netherlands) was prepared as a 0.24 m solution in dry ACN. Coupling time was 2 × 90 s. Oxidizer was prepared as a 0.02 m I_2_ solution in THF/pyridine/H_2_O (70 : 20 : 10, w/v/v/v). Capping reagent A was THF/lutidine/acetic anhydride (8 : 1 : 1) and capping reagent B was 16% *N*‐methylimidazole/THF. RNA>p were synthesized DMT‐off. Detritylations of 3′‐hydroxyl RNAs were performed using 3% dichloroacetic acid in dichloromethane. Oligonucleotides were cleaved from the solid support and the protecting groups on the exocyclic amino groups and the backbone were removed using a 1 : 1 mixture of 40% aqueous methylamine and 25% aqueous ammonia for 1 h at 65 °C. For regular RNA, 2′‐*O*‐TBDMS groups were removed using a mixture of *N*‐methyl‐2‐pyrrolidone (120 µL), triethylamine (TEA; 60 µL) and TEA.3HF, (80 µL) at 70 °C for 2 h. For RNA>p, desilylation and simultaneous cyclization was performed using the same desilylation mixture but with a contact time of 6 h at 40 °C. Desilylation was quenched with trimethylethoxysilane (400 µL), then diisopropyl ether (200 µL) was added to precipitate the oligonucleotide. The precipitate was dissolved in H_2_O and 3′‐OH RNA was purified DMT‐on on an Agilent 1200 series HPLC fitted with a Waters XBridge Oligonucleotide BEH C18 column (10 × 50 mm, 2.5 μm) at 65 °C. Fractions were pooled, dried in a SpeedVac and treated for 15 min with 40% acetic acid at room temperature. After drying in a SpeedVac, oligonucleotides were dissolved in H_2_O and subjected to a DMT‐off purification on RP‐HPLC. RNA>p were purified using only the DMT‐off gradient. Gradient for DMT‐on purification: 10–50% eluent B in 5 min, flow rate = 5 mL·min^−1^. DMT‐off purification: 2–20% eluent B in 8 min, flow rate = 5 mL·min^−1^. Eluent A was 0.1 m triethylammonium acetate, pH 8.0. Eluent B was ACN. The integrities of purified oligonucleotides were confirmed by LC‐MS analysis on an Agilent 1200/6130 system fitted with a Waters acquity UPLC OST C‐18 column (2.1 × 50 mm, 1.7 μm) at 65 °C, with a gradient of 5–35% eluent B in 14 min with a flow rate of 0.3 mL min^−1^. Eluent A was aqueous hexafluoroisopropanol (0.4 m) containing triethylamine (15 mm). Eluent B was methanol.

### Dual‐luciferase reporter assays

Reporter assays were performed in 96‐well plates in HEK293T cells. 8000 cells/ well were seeded in 80 µL Dulbecco's Modified Eagle Medium supplemented with 10% FBS. RNAs were transfected in Opti‐MEM (Invitrogen, Carlsbad, CA, USA) serum‐free medium 8 h after seeding using Lipofectamine 2000 according to the manufacturer’s instructions. Final RNA concentrations were 0, 2.5, 10 and 40 nm. Mock‐treated samples were treated with Opti‐MEM only. Sequences of the positive (siRenilla) and negative controls (siCon) are listed in Table S1. PsiCHECK‐2 reporter plasmids (Promega, Madison, WI, USA; insert sequences listed in Table S2) were transfected 24 h after RNA transfection with jetPEI (Polyplus Transfection) at a final concentration of 40 ng per well according to the manufacturer's instructions. Each replicate experiment was performed in technical triplicates. Mean ± SD are given from three biological replicates. Statistical significance to 0 nm treatment was calculated by two‐way ANOVA and Dunnett’s *post hoc* test. *P < 0.05, **P < 0.01, ***P < 0.001, ****P < 0.0001.

### Isotope‐labeling study

RtcB ligation was performed at 37 °C for 1 h in the presence of 50 mm Tris/HCl (pH = 8), 1 mm MnCl_2_, 0.1 mm GTP, 0.75 µm RtcB, and 0.5 µm RNA>p. Reaction media were prepared in either H_2_^16^O or H_2_^18^O (catalog no 329878; Sigma‐Aldrich). Ligation was quenched through the addition of Na_2_EDTA to a final concentration of 50 mm. LC‐MS analysis was performed on an Agilent 1200/6130 system fitted with a Waters acquity UPLC OST C‐18 column (2.1 × 50 mm, 1.7 μm) at 65 °C, with a gradient of 5–35% eluent B in 14 min with a flow rate of 0.3 mL min^−1^. Eluent A was aqueous hexafluoroisopropanol (0.4 m) containing triethylamine (15 mm). Eluent B was methanol.

### Ligation assay in HeLa lysate

#### HeLa lysate preparation

HeLa cells were cultured in 10 cm^2^ dishes (Techno Plastic Products, Trasadingen, CH) until 90% confluency was reached. Cells were washed with PBS (Gibco, Life Technologies, Invitrogen, Paisley, UK) and lysed by scraping on ice using 400 µL lysis buffer (30 mm HEPES KOH, pH 7.4, 5 mm MgCl_2_, 100 mm KCl, 1× cOmplete EDTA‐free protease inhibitor, 1 mm DTT, 10% glycerol, and 0.5% NP‐40). Cell extracts were collected in 1.5 mL microcentrifuge tubes and incubated on ice for 10 min. Samples were vortexed frequently to ensure efficient lysis. Cell extracts were centrifuged at 17 000 ***g*** at 4 °C for 10 min, and the supernatant was snap‐frozen in 50 µL aliquots. Aliquots were stored at −80 °C. Cell extracts were standardized to a protein concentration of 2.1 mg·mL^−1^. Protein concentration was measured according to the Bradford assay standard procedure for microtiter plates (sample: dye = 1 : 20) as described by the manufacturer (protein assay dye reagent concentrate; Bio‐Rad, Hercules, CA, USA). In brief, 10 µL of cleared cell supernatants of BSA protein standards was mixed in a 96‐well plate with 200 µL diluted dye reagent (1 : 5 dilution in H_2_O). The plate was shaken for 30 s and incubated at room temperature for 15 min. Absorption was measured at 620 nm for 1 s per well.

#### ^32^P radiolabeling

RNAs were radioactively labeled (15 µCi) using either T4‐polynucleotide kinase (10 000 U·mL^−1^) for 3′‐hydroxyl RNAs or T4 PNK (3′‐phosphatase minus; 10 000 U·mL^−1^; both from New England Biolabs, Ipswich, MA, USA) for RNA>p and [γ‐32P] adenosine triphosphate (ATP, 6000 Ci·mmol^−1^, 150 mCi·mL^−1^; Perkin Elmer, Waltham, MA, USA). One microlitre 1 of RNA probe (100 nm final) was mixed with RNAsin, RNase‐free water, PNK buffer, T4 PNK, and [γ‐32P] ATP in a dedicated Type C laboratory and incubated at 37 °C for 30 min followed by 5 min of heat inactivation at 95 °C. Probes were purified using G‐25 sephadex spin columns (GE Healthcare, Chicago, IL, USA).

#### Ligation mixture

Radiolabeled RNA was annealed to different counter‐strands by heating in annealing buffer (30 mm HEPES KOH pH 7.4, 4 mm MgCl_2_, 200 mm KCl) at 95 °C for 2 min and cooled down to room temperature over 3 h. Where indicated, a bridging ORN (complementary to the 3′ end of miR‐34a‐5p>p and the 5′‐end of the counter‐strand) was added in 10‐fold excess. Two microlitre 40 nm annealed RNA (8 nm final) was mixed with 4 μL HeLa extract (protein conc. 2.1 mg·mL^−1^) and 4 μL buffer [100 mm KCl, 3 mm MgCl_2_, 12.5 mm DTT, 7.5 mm ATP, 0.5 mm GTP, 1 U·µL^−1^ RNasin (Promega)]. Ligation mixtures were incubated at 30 °C for 30 min. Ligation was quenched through the addition of 150 μL proteinase K solution (diluted to 1.8 mg·mL^−1^; Roche, Basel, Switzerland, catalog no 3115828001) and incubation at 65 °C for 15 min. Samples were mixed with 10 μL 2× formamide loading dye (95% formamide, 0.005% bromphenol blue, 0.05% xylene cyanol FF, 4 mm EDTA) and heated at 95 °C for 5 min. Samples (15 μL) were loaded on a 15% denaturing polyacrylamide gel (dPAGE) and analyzed in 0.5× TBE buffer at 25 mA for 4 h. The experiment was performed in three independent replicates.

### RtcB ligation assays

One microlitre 10 μm annealed RNA duplexes (0.5 μm final) were mixed with 2 μL 10× RtcB reaction buffer (New England Biolabs), 1 μL 15 μm RtcB (0.75 μm final; New England Biolabs, M0458S), 2 μL 1 mm GTP, 2 μL 10 mm MnCl_2_ and 12 μL RNase‐free H_2_O (total volume = 20 μL) and incubated at 37 °C for 1 h. Samples were analyzed by 15% dPAGE and visualized with SYBR Gold Nucleic Acid Gel Stain (Thermo Fisher).

## Results

In this study, we aimed to investigate enzymatic ligation as an alternative to cross‐linking in order to capture miRNA‐mRNA interactions, using a new class of miRNA mimics functionalized with 2′, 3′‐cyclic phosphate groups (miRNA>p; Fig. 1C). We hypothesized that ligation could occur between the cyclic phosphate and free 5′‐OH groups of bound target mRNAs in analogous fashion to chimera formation during CLASH and CLEAR‐CLIP methods. Furthermore, we postulated that the conversion efficiency would be high since all miRNA probes carry the cyclic phosphate group introduced during synthesis.

We recently described the preparation of short 2′, 3′‐cyclic phosphate‐terminated RNAs using standard phosphoramidite chemistry on a novel, dedicated CPG solid support [41, 42, 43] (Fig. S1). Using this approach, we synthesized and purified miRNA>p probes based upon three miRNAs that play prominent roles in cellular mechanisms: miR‐34a, miR‐106a and let‐7 (Fig. S2, Table 1). To help demonstrate the robustness of this chemistry, we also synthesized a structured RNA derived from a miRNA precursor (pre‐20>p) and a 64‐nt‐long RNA (ORN_64_>p).

**Table 1:** 2′, 3′‐cyclic phosphate‐terminated RNAs synthesized in this study.

Name	Sequence (5′–3′)	Mass calc.	Mass found
miR‐34a>p	UGGCAGUGUCUUAGCUGGUUGU>p	7091.16	7090.45
miR‐106a>p	AAAAGUGCUUACAGUGCAGGUAG>p	7496.53	7495.58
let‐7g>p	UGAGGUAGUAGUUUGUACAGUU>p	7123.21	7123.50
pre‐20>p	UGCAGGUAGUUUUGGCAUGACUCUACUGUA>p	9630.71	9629.91
ORN_64_>p	UCAAGAGACUAAAUCCUUCCAUGAAUAGUUUUUCAAGAGAAAACUAUUCAUGGAAGGAUUUAGU>p	20 568.35	20 567.40

**Fig. 2:**
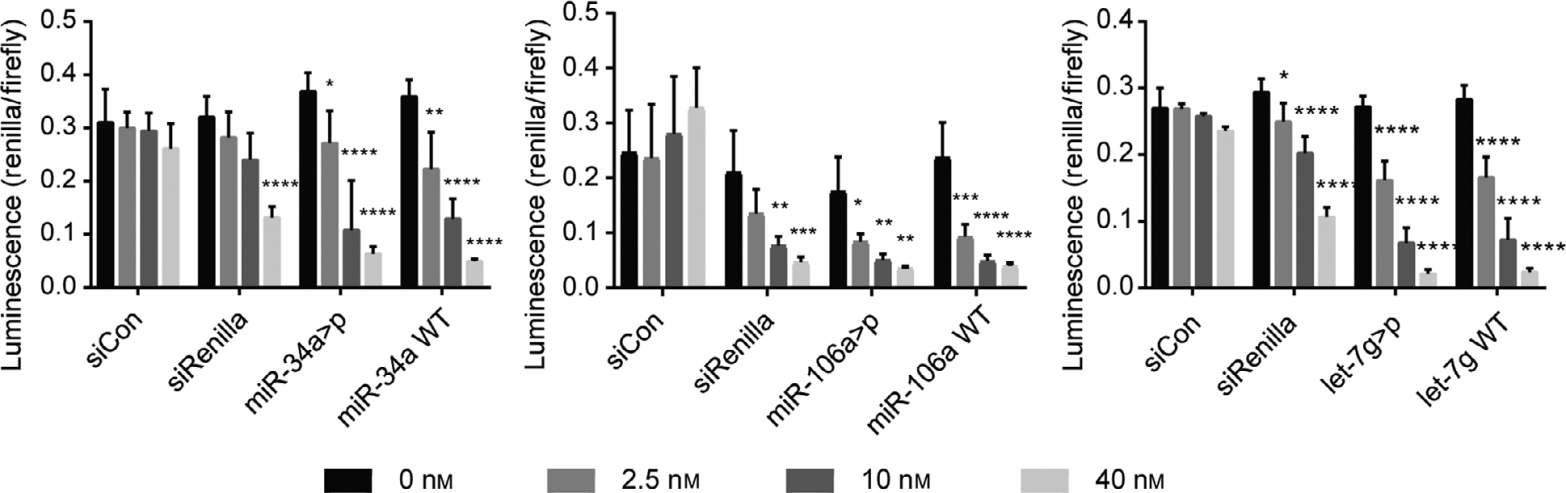
miRNA>p probes are taken into miRISC and suppress their targets. Activity of miR‐34a>p, miR‐106a>p and let‐7g>p in dual‐luciferase reporter assays in HEK293T cells (2′,3′‐cycP on 5p strands). Increasing RNA concentrations from left to right: 0, 2.5, 10, and 40 nm. Assays were performed with psiCHECK‐2 plasmids containing fully complementary target sites to the miRNA guide (three sites for miR‐34a‐5p; one site for miR‐106a‐5p and let‐7g‐5p). Cyclic phosphate‐terminated 5p‐strands (Table 1) were annealed to the corresponding 3p‐strands (Table S1) prior to transfection. Mean ± SD shown from three independent experiments, each performed in technical triplicates. Asterisks indicate statistical significance to 0 nm treatment calculated by two‐way ANOVA and Dunnett’s *post hoc*test. *P < 0.05, **P < 0.01,*** P < 0.001,**** *P* < 0.0001. WT = wild‐type (3′‐OH terminus).

In order to capture their cellular targets, functionalized miRNAs have to be accepted into miRISC. In principle, any structural modification to a miRNA might influence its binding to, and suppression of mRNA targets. Therefore, we conducted two types of cell assays to determine whether the 3′ cyclic phosphate group would affect target recognition in miRISC. Thus, dual‐luciferase reporter plasmids containing fully complementary target sites to each of miR‐34a, miR‐106a and let‐7g were prepared (Table S2). These were transfected into HEK293T cells prior to treatment with graded concentrations of RNA duplexes comprising the miRNA>p probes, the corresponding wild‐type miRNAs and controls. The three miRNA>p's inhibited their targets with a similar efficiency to unmodified wild‐type miRNA mimics, confirming their uptake into miRISC and the activation of the modified guide strand for target engagement (Fig. 2). In a second approach, we compared the effects of wild‐type miR‐106a and miR‐106a>p on the levels of five native mRNAs (ZNFX1, ANKRD52, FAM102A, MINK1, CDKN1A) that are either known or predicted to be regulated by miR‐106a after transfection in HEK293T cells. Both reagents produced identical effects on these targets, strongly inhibiting CDKN1A and ANKRD52 to the same degree, and having no activity on the remaining predicted targets (Fig. S3). Taken together, the outcome of these experiments provides strong evidence that cyclic phosphate modified miRNAs recognize native targets similarly to their parent wild‐type miRNAs.

In canonical interactions, base‐pairing between a miRNA and its mRNA target is strongest in the miRNA seed region, and weak or nonexistent at the 3′end [1, 44, 45]. Consequently, a given miRNA may bind to hundreds of different target mRNA sequences [45]. In addition, the nuclease trimming step during a CLIP protocol cleaves miRNA‐bound and RISC‐protected mRNAs in an incomplete and heterogeneous fashion (Fig. 1A). Therefore, the ligation via the cyclic phosphate is required to be insensitive to the nature (length, composition, and complementarity) of the hanging ends of the two strands (Fig. 1C). In an effort to identify a general set of assay conditions that would produce efficient ligations in miRISC for the widest possible range of miRNA sequences and their targets, we performed model experiments in which we examined how ligation efficiency varies with the degree of complementarity between the two RNA strands.

**Fig. 3:**
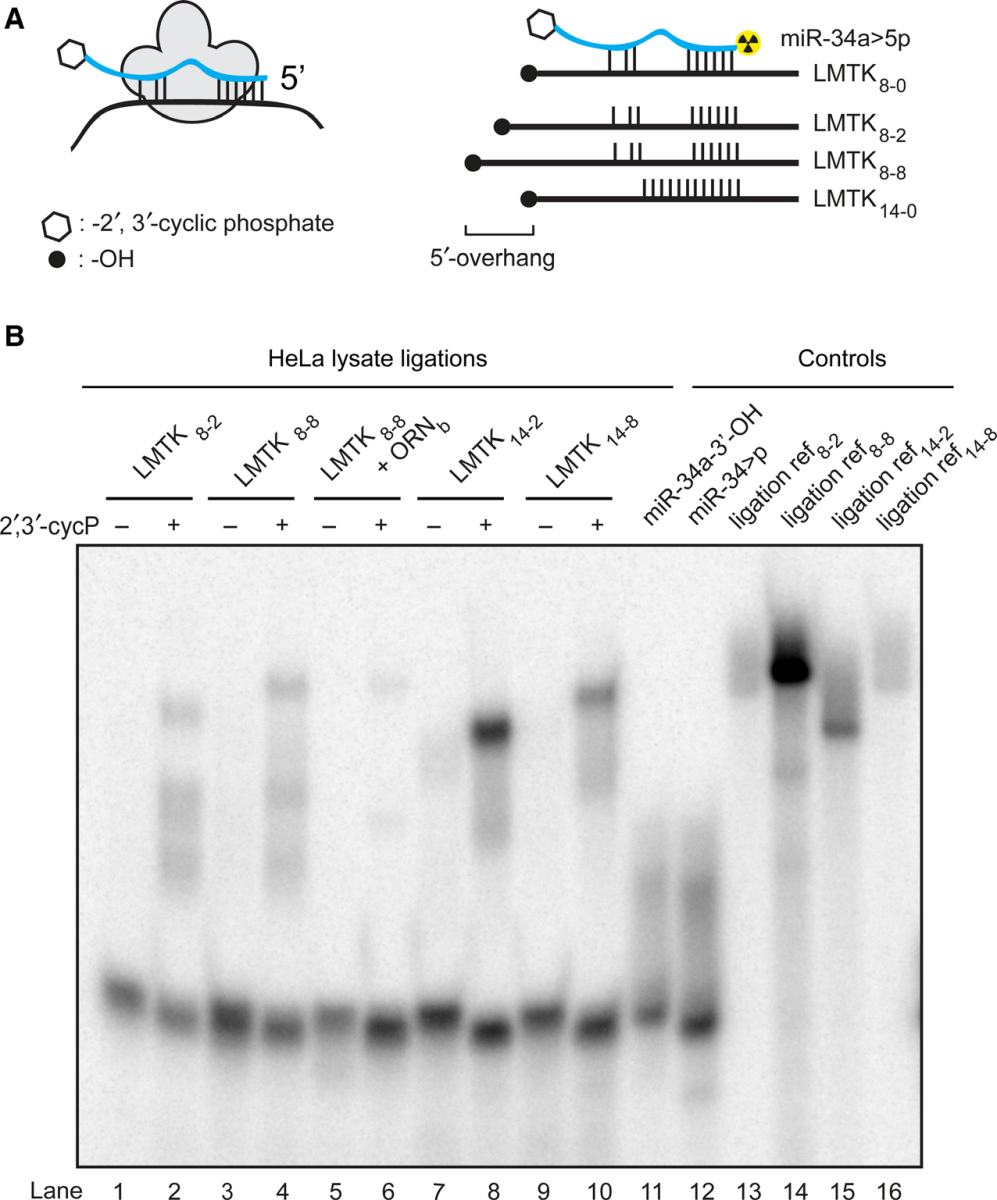
Ligation of 2′, 3′‐cyclic phosphate miR‐34a‐5p strands to 5′‐OH mRNA mimics in HeLa lysate. (A) Scheme showing principle and different formats of RNA>p ligation. Left: miRNA>p probe bound to a target mRNA inside the RNA‐induced silencing complex (RISC). Right: Alignments of radiolabeled miR‐34a‐5p to LMTK3‐mRNA mimics. (B) Ligation of miR‐34a‐5p>p (22 nt) to different LMTK3 mRNA mimics (64–72 nt) in HeLa lysate. Mir‐34a‐5p>p ligation is shown in lanes 2, 4, 6, 8, and 10. Controls are shown in lanes 11–16. Mir‐34a‐5p>p (2′,3′ cycP +) and miR‐34a‐5p‐3′‐OH (2′,3′ cycP ‐) were 5′‐^32^P labeled using T4 PNK (3′‐phosphatase minus). Mir‐34a‐5p>p was annealed to different RNA counter‐strands and mixed (8 nmfinal) with HeLa extract (protein conc. = 2.1 mg·mL^−1^; 0.84 mg·mL^−1^final) and a ligation buffer containing KCl (40 nmfinal), EDTA (pH = 8, 100 µmfinal), MgCl_2_(1.2 mmfinal), DTT (5 mmfinal), ATP (3 mmfinal), GTP (0.2 mmfinal), and RNasin (1 U·µL^−1^; Promega) and incubated at 37 °C for 30 min. In one setup, a bridging ORN_b_(30 nm, 12 nt) complementarity to each, mir‐34a‐5p>p and LMTK‐3 mimic_8‐8_, was added prior to annealing. Reference oligonucleotides for the respective ligation products (Controls) were chemically synthesized; differences in band intensity may be caused by differences in labeling efficiency. Representative gel shown,*n* = 3. Full gel shown in Fig. S5.

MiR‐34a is a prominent miRNA that is regulated by p53; it broadly influences gene expression and mediates apoptosis in cells [46, 47, 48]. We investigated ligation of miR‐34a>p to counter‐strands with various lengths of 5′‐overhang to understand how heterogeneous trimming may affect the efficiency of ligation. For these experiments, we selected an experimentally validated target of miR‐34a, the mRNA of the *LMTK3* gene [49, 50]. The 5p‐strand of miR‐34a>p was 5′‐labeled with a ^32^P phosphate (Fig. 3) using T4 PNK (3′‐phosphatase minus to avoid hydrolysis of the cyclic phosphate) and we examined its ligation in HeLa lysates with four counter‐strand sequences comprising either 8 or 14 nt of complementarity to the seed region of the miRNA>p, and a 5′‐overhang of either two or eight nt on the LMTK3 model sequences (LMTK_8‐2_, **LMTK_8‐8,_** **LMTK_14‐2_** and **LMTK_14‐8_**; Figs 3A, Fig. S4). We used 14‐nt of base‐pairing to ensure quantitative hybridization between the strands in the assay, whereas in the actual protocol the strands would be stabilized as a duplex by miRISC. Reference oligonucleotides for the respective ligation products (Controls) were chemically synthesized and included as controls. Importantly, no exogenous ligase was added to the crude HeLa extracts in this experiment.

The 5p‐strand of miR‐34a>p and the counter‐strands were incubated in HeLa lysates and after work‐up, radiolabeled RNAs were visualized on dPAGE. The presence of new, slower‐migrating bands matching the migration of the expected ligation products (lengths 64–72 nt; lanes 13–16, Fig. 3B) implied successful ligation of the probe to the respective counter‐strands (lanes 2, 4, 6, 8, 10; Fig. 3B). The formation of additional bands, which migrate faster than the ligation products from miR‐34a>p and LMTK3 counter‐strands (lanes 2, 4, 8, 10; Fig. 3B), could have been due to homo‐ligation between the labeled and unlabeled fraction of miR‐34a>p, or due to ligation to other RNAs present in the HeLa extract. In general, the presumed product band was more intense for the duplexes with higher complementarity (14 nt; lanes 8, 10; Fig. 3B) and with the 2‐nt overhang (**LMTK_14‐2_**, lane 8, Fig. 3B). Importantly, formation of the presumed product required the cyclic phosphate group and was not present in the gel with 3′‐hydroxyl RNAs (lanes 1, 3, 5, 7, 9; Fig. 3B). Furthermore, the data suggest that the miRNA>p's are sufficiently stable against endogenous cyclic nucleotide phosphatases to participate in ligation reactions, at least in HeLa cell lysates. Nevertheless, ligation was rather inefficient, particularly for the model targets with lower complementarity (LMTK_8‐2_, **LMTK** **_8‐8_)**. Addition of a bridging oligoribonucleotide **ORN_b_** with equal complementarity to both sides of the RNA junction greatly reduced ligation efficiency (lanes 5, 6; Fig. 3B).

The results observed for these model substrates suggested that this protocol was not optimal to ensure efficient ligations for a wide range of miRNA‐mRNA sequences, that is, in sequence‐independent fashion. Therefore, we turned to the use of a recombinant ligase that could be added during the miR‐CLIP protocol to provide homogeneous ligation in sequence‐independent fashion.

**Fig. 4:**
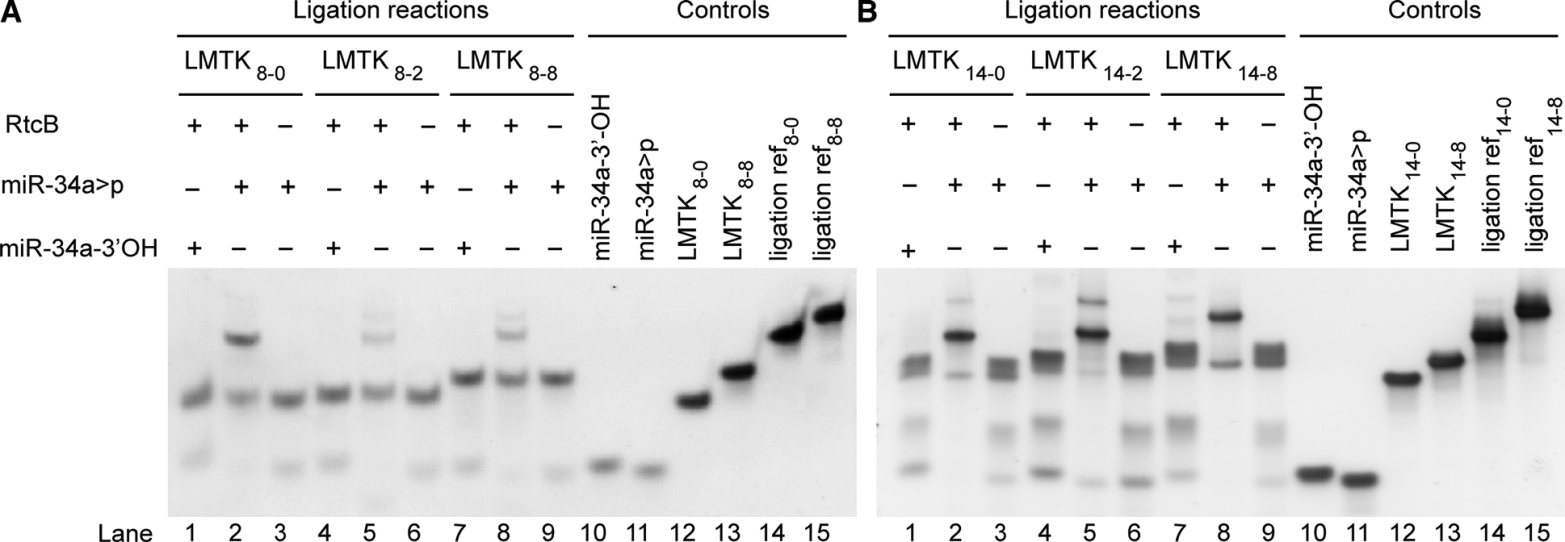
RtcB‐mediated ligation of 2′, 3′‐cyclic phosphate miR‐34a‐5p strands to 5′‐OH mRNA mimics. (A) RtcB‐mediated ligation of miR‐34a‐5p>p (22 nt) to different LMTK3 mRNA mimics (64–72 nt) with 8 consecutive and (B) 14 consecutive complementary nt and a variable 5′ overhang of the LMTK3 counter‐strands (0, 2 or 8 nt overhang). Reaction mixtures contained 0.5 μm RNA duplexes, 0.75 μmRtcB, 0.1 mm GTP, 1 mm MnCl_2_, and 1x RtcB reaction buffer. Reference oligonucleotides for the respective ligation products (Controls) were chemically synthesized. Staining with SYBR Gold Nucleic Acid Gel Stain, *n* = 3, representative gels shown. Full gels shown in Fig. S6.

Thus, we evaluated ligation of miR‐34a>p with the same counter‐strands in a lysate‐free buffer system after the addition of recombinant RNA>p/3′‐p‐ligase RtcB (Figs 4 and S6). Once again, appearance of bands that were consistent with the formation of ligated products was dependent on the presence of the cyclic phosphate group as opposed to 3′‐hydroxyl ends. Ligation efficiency was highest for the target sequence with the shortest overhang in the 8 nt complementarity series (**LMTK_8_** series; lane 2; Figs 4A and S6a, S7a). For the duplexes with higher complementarity (**LMTK_14_** series), a generally higher ligation efficiency, independent of overhang length (lanes 2, 5, 8; Figs 4B and S6b, S7b), was observed. In some samples (lanes 2, 5; Fig. 4B) we observed small amounts of an additional, slower‐migrating band, that may have been the result of further ligation of the chimeric product to additional miR‐34a>p. In general, RtcB‐mediated ligation of miRNA>p probes was more efficient and ‘cleaner’ than ligations performed in crude HeLa extracts (Fig. 3) We concluded that RtcB‐mediated ligation would provide a more robust ligation with lower variabilities across sequences and cell types.

**Fig. 5:**
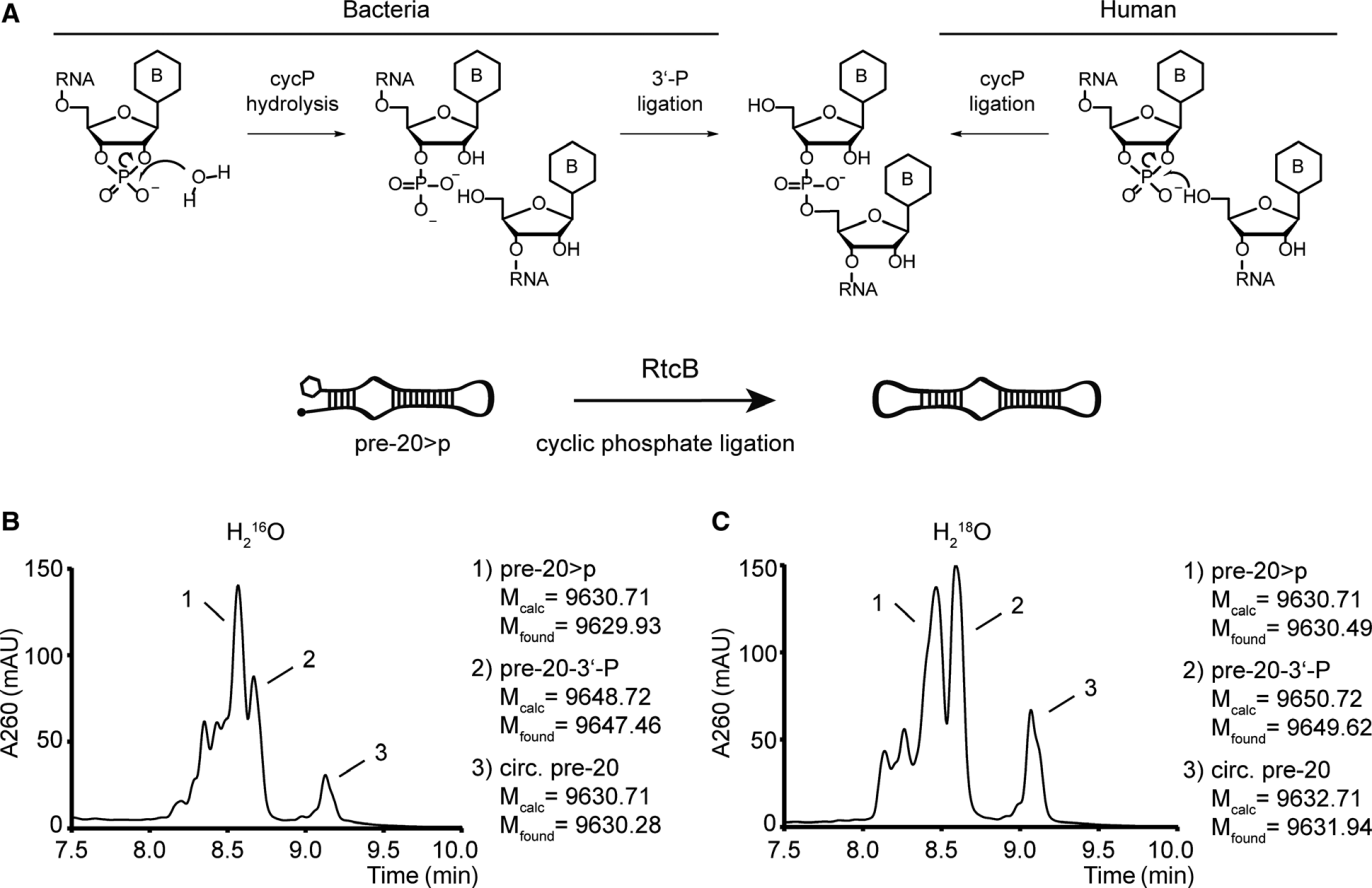
Mechanistic details of RtcB‐mediated RNA>p ligation (A) Different mechanisms suggested for bacterial and human RNA>p ligation. Direct ligation between RNA 5′‐OH and 2′, 3′‐cyclic phosphate termini has been suggested in HeLa lysate [19, 37, 38]. For bacterial RtcB, hydrolysis of the 2′, 3′‐cycP to a 3′‐P terminus and subsequent ligation of the 3′‐P terminus has been suggested [29, 30, 31, 32, 33, 34, 35, 36]. (B) RtcB‐mediated cyclization of the stem‐loop RNApre‐20>pin H_2_^16^O and (C) H_2_^18^O. RtcB ligation was performed at 37 °C for 1 h in the presence of 50 mm Tris/HCl (pH = 8), 1 mm MnCl_2_, 0.1 mmGTP, 0.75 µm RtcB and 0.5 µm pre‐20>p. Ligation was quenched through the addition of Na_2_EDTA to a final concentration of 50 mm. LC‐MS analysis was performed on an Agilent 1200/6130 system fitted with a Waters acquity UPLC OST C‐18 column (2.1 × 50 mm, 1.7 μm) at 65 °C, with a gradient of 5–35% eluent B in 14 min with a flow rate of 0.3 mL·min^−1^. Eluent A was aqueous hexafluoroisopropanol (0.4 M) containing triethylamine (15 mm). Eluent B was methanol. UV trace (260 nm) between 7.5 and 10 min is shown. B = nucleobase.

Previous work conducted on a mammalian ligase in HeLa lysate [37, 38, 51] as well as thermodynamics considerations [30] have suggested that RtcB‐mediated RNA>p ligation may proceed through direct attack of an RNA 5′‐hydroxyl group at the 2′, 3′‐cyclic phosphate group [19, 37, 38, 51]. On the other hand, a series of mechanistic studies on recombinant RtcB rather indicated a multistep process in which the cyclic phosphate is first opened to a 3′‐monophosphate, followed by activation as a GMP‐adduct and ligation from attack of a 5′‐hydroxyl group (Fig. 5A) [29, 30, 31, 32, 35, 36]. To clarify how this unusual ligation mechanism proceeded with the miRNA>p's in this study, we performed an isotope‐labeling study in which RtcB‐dependent cyclization of a stem‐loop RNA derived from a miRNA precursor (pre‐20>p) was performed in H_2_^16^O and H_2_^18^O (Figs 5 and S9, S10). In the event of a direct attack of the 5′‐OH RNA on the cyclic phosphate, the molecular weight of the ligation product should be unaffected by the isotopic composition of the reaction solvent. On the other hand, a multistep pathway should lead to the incorporation of some ^18^O at the ligation site. We first tested the suitability of the LC‐MS system to detect incorporation of ^18^O at a single phosphodiester linkage. For this purpose, we synthesized a short RNA sequence (**ORN_t_**) with standard ^16^O phosphodiester linkages and a single ^18^O at the terminal site. The observed difference of 1.92 Da in the product masses provided confidence that the assay could distinguish hydrolysis‐dependent ^18^O incorporation (Fig. S8). We hence performed RtcB‐mediated cyclization of pre‐20>p in H_2_^16^O and H_2_^18^O. Ligation was quenched after 1 h by addition of EDTA to remove Mn^2+^ and was then analyzed by LC‐MS. Cyclization of the RNA was confirmed by the appearance of a second signal (peak#3) with the same mass in the chromatogram but with an extended retention time over the linear starting material (peak#1), consistent with earlier studies on cyclic phosphate‐containing oligoadenylates (Fig. 5B) [41]. However, the observed mass of the ligation product for the reaction in H_2_^18^O was increased by 1.45 Da compared to the remaining RNA>p and by 1.66 Da compared to the corresponding ligation product under H_2_^16^O conditions (Fig. 5B,C). Together with the appearance of a signal (peak#2) corresponding to the respective 3′‐phosphate intermediate, these data provide further evidence for the multistep pathway that proceeds with hydrolysis of the cyclic phosphate followed by ligation of the 3′‐phosphate intermediate [29, 30, 31, 32, 35, 36]. The data were confirmed in a second experiment in which the excess Mn^2+^ was not depleted prior to LC‐MS analysis (data not shown).

## Discussion

Delineating interactions between miRNAs and their targets is key to expanding our knowledge about the wide variety of functions of miRNAs, including the molecular basis of many diseases [45, 52]. A variety of CLIP methods exist to identify the targets of miRNAs, each with their strengths and weaknesses [7, 8, 9, 10, 11, 14, 15, 16, 40]. A subgroup of these methods involves ligation of miRNAs to their binding partners and thereby provides information on the precise binding sites of the miRNAs [8, 9, 14, 15, 40]. For this purpose, immunopurified RNAs are 5′‐phosphorylated and joined through the addition of T4 RNA ligase. Nevertheless, despite efforts to optimize this chemistry [8, 9, 14, 15, 40], the ligation efficiency remains low and limits the rate of chimera formation [16]. Interestingly, in some cases truncated chimeras were obtained in the absence of an exogenously added ligase. These were thought to result from endogenous ligation of RNA>p species, originating from the nuclease‐mediated degradation of the original miRNA [14, 40]. This conclusion is supported by the finding that the miRNA targetome identified from these truncated chimeras matched the targets that had been recovered using a dedicated miRNA/target RNA ligation step [14, 40].

We are presently investigating novel methods of enzymatic ligation as a means to capture miRNA‐mRNA interactions, using a new class of miRNA mimics functionalized with 2′, 3′‐cyclic phosphate groups (miRNA>p). These mimics performed as native miRNAs in cells. We hypothesized and showed that ligation could occur between the cyclic phosphate and free 5′‐OH groups of bound target mRNAs, which are produced after a nuclease trimming step in the CLIP protocol. Furthermore, we postulated and demonstrated that high ligation efficiencies can be expected from miRNA>p probes, since they all carry the cyclic phosphate group introduced during synthesis. This differs from a conventional CLIP protocol, where the captured RNA population undergoes a separate phosphorylation treatment of unknown and variable efficiency prior to ligation (e.g., by T4 RNA ligase I).

Here, we have investigated the use of recombinant RtcB to ligate the ends of partially complementary RNAs in model experiments that mimic miRNA‐mRNA duplexes in miRISC after nuclease trimming. We found that RtcB‐mediated ligations were more efficient than ligations performed by endogenous ligases in HeLa cells. We also confirmed that the multistep RtcB mechanism proceeded *via* a terminal monophosphate intermediate [29, 30, 31, 32, 35, 36]. This implies that 3′‐phosphorylated miRNAs might also function as probes for the new CLIP method using RtcB. Hence, ligation is ensured, even in the event of any partial hydrolysis that may occur, for example in different cell types. Overall, the results of the study serve as a solid basis for the use of chemically synthesized RNA>p species as probes for an improved CLIP method to discover the targetomes of miRNAs.

## Author contributions

CB and YW contributed equally. CB designed the study, performed experiments, analyzed data, and drafted the paper. YW designed the study, performed experiments, analyzed data, and drafted the paper. AL and ST performed experiments and analyzed data. JH designed the study and drafted the paper.

## Supporting information


**Fig. S1.** Chemical synthesis of RNA>p using the 2′, 3′‐cyclic phosphate solid support [1, 2].
**Fig. S2.** LC‐MS analysis of purified RNA>p.
**Fig. S3.** MiR‐106a>p suppresses native mRNA targets at the same level as miR‐106a WT.
**Fig. S4.** Sequence alignments of miR‐34a‐5p with the respective LMTK3 counter‐strands.
**Fig. S5.** Full gel corresponding to Fig. 3b.
**Fig. S6.** Full gel corresponding to Fig. 4.
**Fig. S7.** Ratio of intensity ligation product to total lane intensity from Fig. 4.
**Fig. S8.** System suitability test to detect the difference between 16O and 18O containing oligonucleotides.
**Fig. S9.** Full chromatograms and mass spectrometry from Fig. 3.
**Fig. S10.** Deconvoluted ion sets from Figure S9.
**Table S1.** Sequences of oligoribonucleotides used in this study.
**Table S2.** Sequences of DNA inserts for Dual Luciferase reporter plasmids.
