# Supplementary material for: Ligation of 2′, 3′‐cyclic phosphate RNAs for the identification of microRNA binding sites
Source: FEBS Lett. 2020 Nov 16;595(2):230–40. doi: 10.1002/1873-3468.13976 (PMC7894349; doi:10.1002/1873-3468.13976)
Supplement: Supplementary file 1 — Fig. S1. Chemical synthesis of RNA>p using the 2′, 3′‐cyclic phosphate solid support [1, 2]. Fig. S2. LC‐MS analysis of purified RNA>p. Fig. S3. MiR‐106a>p suppresses native mRNA targets at the same level as miR‐106a WT. Fig. S4. Sequence alignments of miR‐34a‐5p with the respective LMTK3 counter‐strands. Fig. S5. Full gel corresponding to Fig. 3b. Fig. S6. Full gel corresponding to Fig. 4. Fig. S7. Ratio of intensity ligation product to total lane intensity from Fig. 4. Fig. S8. System suitability test to detect the difference between 16O and 18O containing oligonucleotides. Fig. S9. Full chromatograms and mass spectrometry from Fig. 3. Fig. S10. Deconvoluted ion sets from Figure S9. Table S1. Sequences of oligoribonucleotides used in this study. Table S2. Sequences of DNA inserts for Dual Luciferase reporter plasmids. [file FEB2-595-230-s001.pdf]

Ligation of 2', 3'-cyclic phosphate RNAs for the identification of microRNA binding sites

Supporting Information

Christian Berk[1‡], Yuluan Wang[1‡], Artur Laski[1], Stylianos Tsagkris[1†] and Jonathan Hall[1*]

[1] Institute of Pharmaceutical Sciences, Department of Chemistry and Applied Biosciences, ETH Zurich, Zurich, Switzerland

[†] Present address: Max Planck Institute of Immunobiology and Epigenetics, Freiburg, Germany

‡ These authors contributed equally to this work

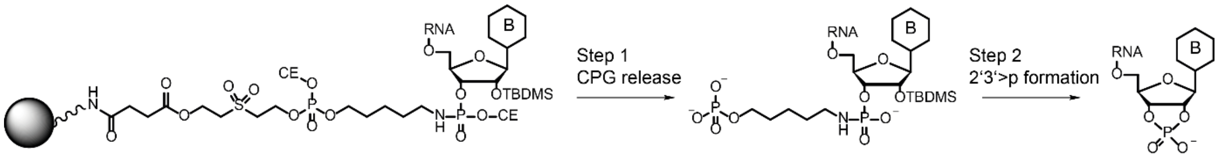

Figure S1. Chemical synthesis of RNA>p using the 2’, 3’-cyclic phosphate solid support[1, 2]. Step 1: Incubation of the solid support-bound RNA with a 1:1 mixture of 40% aqueous methylamine and 25% aqueous ammonia at 65°C for 1 h (AMA). Step 2: Removal of 2’-TBDMS groups and subsequent formation of a 2’, 3’-cyclic phosphate terminus through incubation with a mixture of NMP, TEA and TEA.3HF at 40°C for 6 h. B = nucleobase, CE = cyanoethyl, TBDMS = tert-butyldimethylsilyl. Gray sphere represents the controlled pore glass (CPG) solid support.

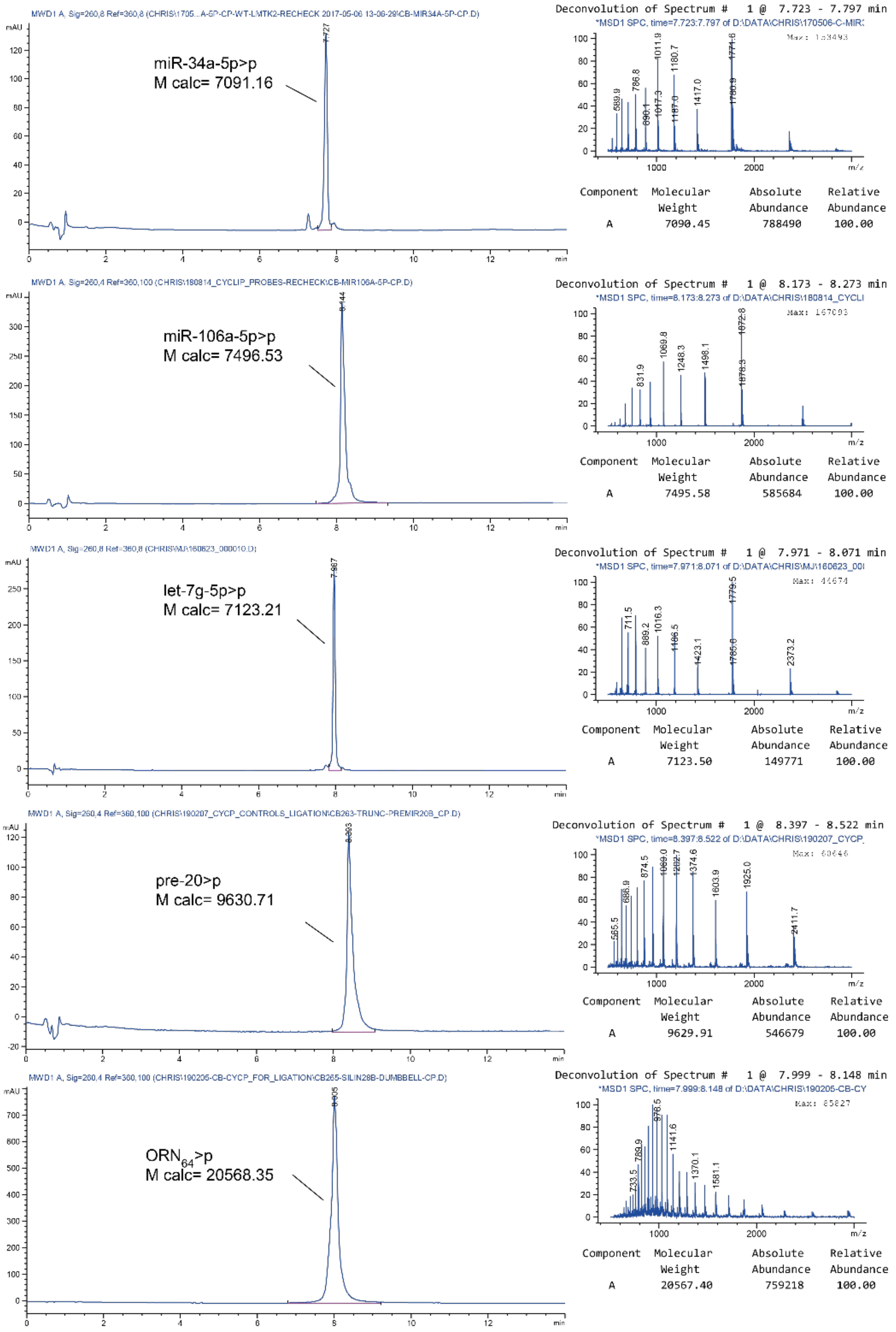

Figure S2. LC-MS analysis of purified RNA>p. Analysis was performed on an Agilent 1200/6130 system fitted with a Waters acquity UPLC OST C-18 column (2.1×50 mm, 1.7 µm) at 65°C, with a gradient of 5-35% eluent B in 14 min with a flowrate of 0.3 mL min^{-1}. Eluent A was aqueous hexafluoroisopropanol (0.4 M) containing triethylamine (15 mM). Eluent B was methanol. UV trace (260 nm) and mass spectra are shown.

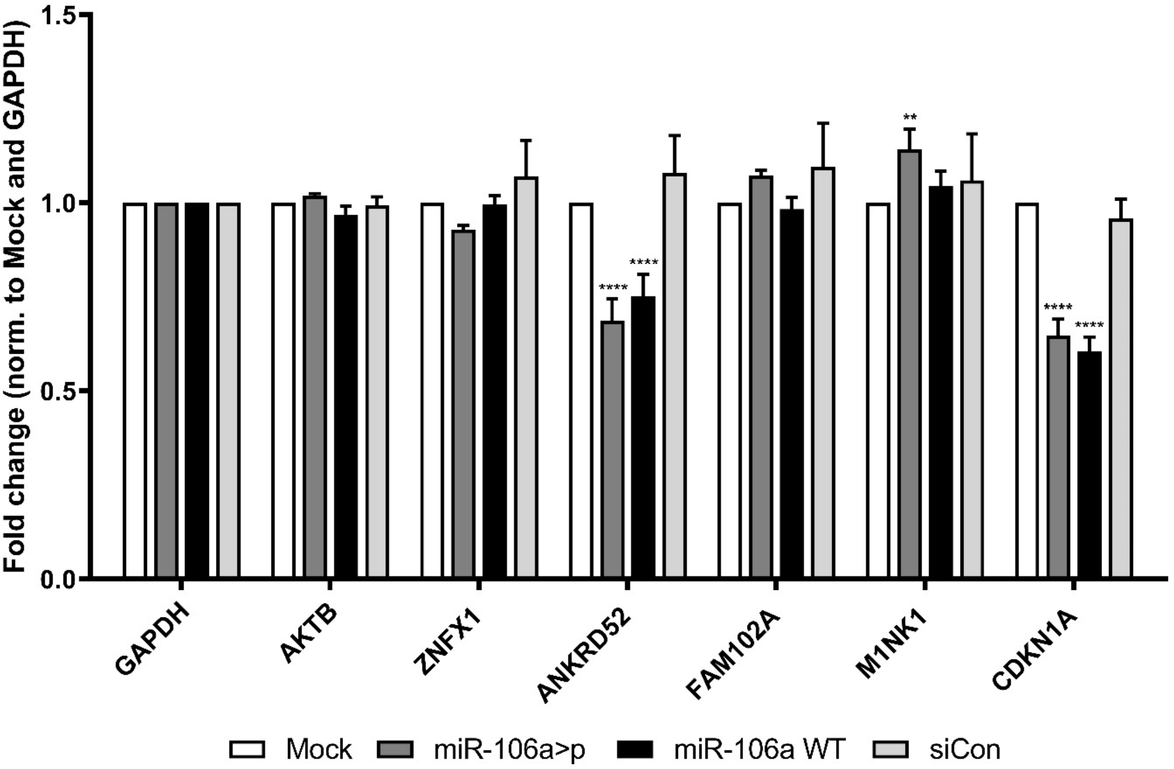

Figure S3. MiR-106a>p suppresses native mRNA targets at the same level as miR-106a WT. Fold change of miR-106a target mRNAs in HEK293T cells following treatment with 40 nM miR-106a>p, miR-106a WT or siCon. Asterisks indicate significance between tested condition and mock treatment. Significance calculated by 2-way ANOVA followed by Dunnett's test: * ($P \leq 0.05$), ** ($P \leq 0.01$), *** ($P \leq 0.001$), **** ($P \leq 0.0001$). N=3, with N=2 for miR-106a>p treatment.

3′-UGUUGGUCGAUUCUGUGACGGU-5′ miR-34a-5p
5′-GUGGAUGACGGCGCCACUGCCACCACCGCAGACGCCGCCUCU-3′ $LMTK_{8-0}$
5′-CUGUGGAUGACGGCGCCACUGCCACCACCGCAGACGCCGCCUCU-3′ $LMTK_{8-2}$
5′-AAUCCUCUGUGGAUGACGGCGCCACUGCCACCACCGCAGACGCCGCCUCU-3′ $LMTK_{8-8}$

3′-UGUUGGUCGAUUCUGUGACGGU-5′ miR-34a-5p
5′-GUGGAUGACUAAGACACUGCCACCACCGCAGACGCCGCCUCU-3′ $LMTK_{14-0}$
5′-CUGUGGAUGACUAAGACACUGCCACCACCGCAGACGCCGCCUCU-3′ $LMTK_{14-2}$
5′- AAUCCUCUGUGGAUGACUAAGACACUGCCACCACCGCAGACGCCGCCUCU-3′ $LMTK_{14-8}$

Figure S4. Sequence alignments of miR-34a-5p with the respective LMTK3 counter-strands. Complementary nucleotides are highlighted in yellow.

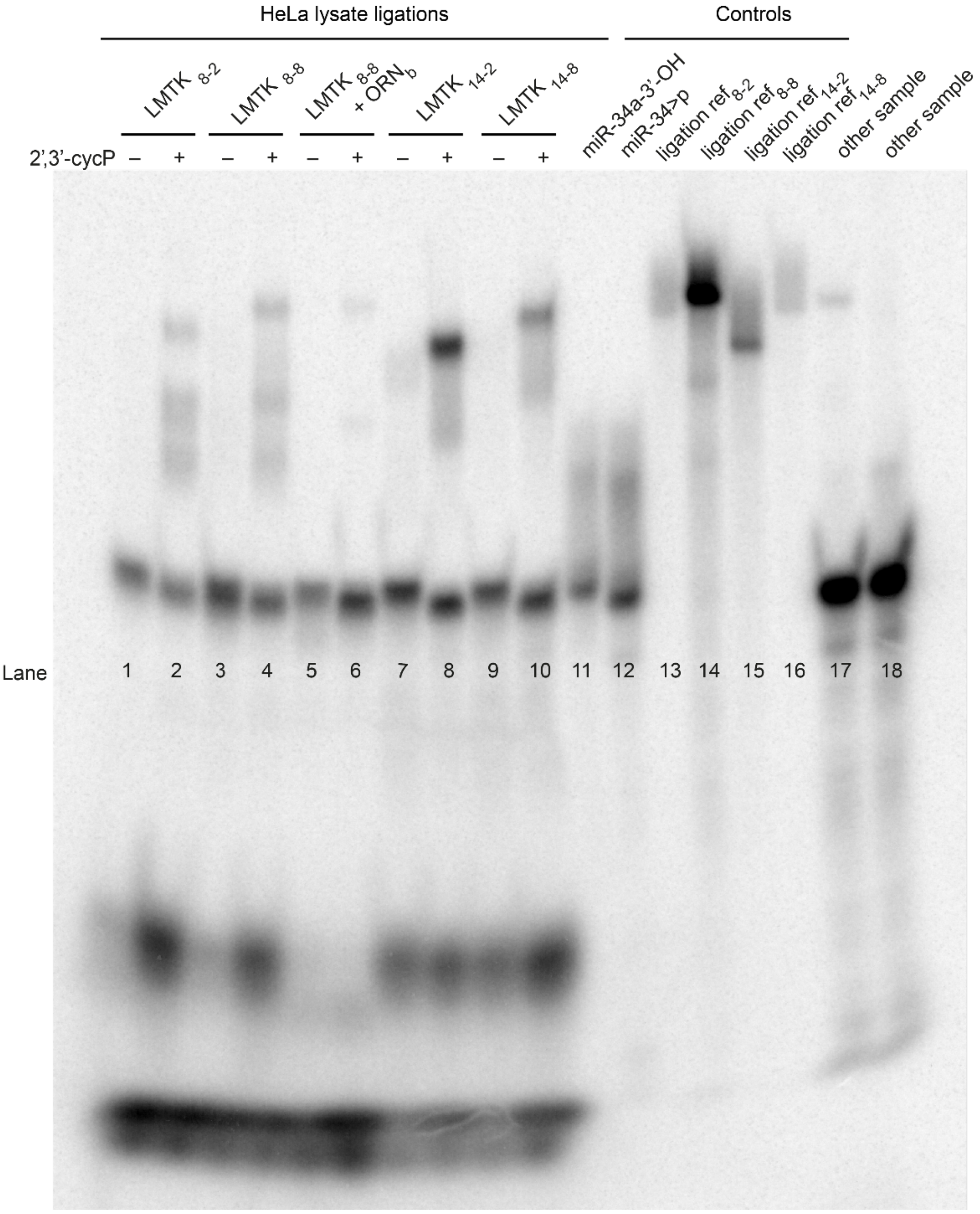

Figure S5. Full gel corresponding to **Fig. 3b**. Ligation of miR-34a-5p>p (22 nt) to different LMTK3 mRNA mimics (64-72 nt) in HeLa lysate. Mir-34a-5p>p ligation is shown in lanes 2, 4, 6, 8, 10. Controls are shown in lanes 11-16. Mir-34a-5p>p (2',3' cycP +) and miR-34a-5p-3'-OH (2',3' cycP -) were 5'-^{32}P labelled using T4 PNK (3'-phosphatase minus). Mir-34a-5p>p was annealed to different RNA counter-strands and mixed (8 nM final) with HeLa extract (protein conc. = 2.1 mg/ml; 0.84 mg/ml final) and a ligation buffer containing KCl (40 nM final), EDTA (pH = 8, 100 µM final), $MgCl_2$ (1.2 mM final), DTT (5 mM final), ATP (3 mM final), GTP (0.2 mM final) and RNasin (1 U/µl, Promega) and incubated at 37°C for 30 min. In one setup, a bridging ORN_b (30 nM, 12 nt complementarity to each, mir-34a-5p>p and LMTK-3 $mimic_{8-8}$, was added prior to annealing.

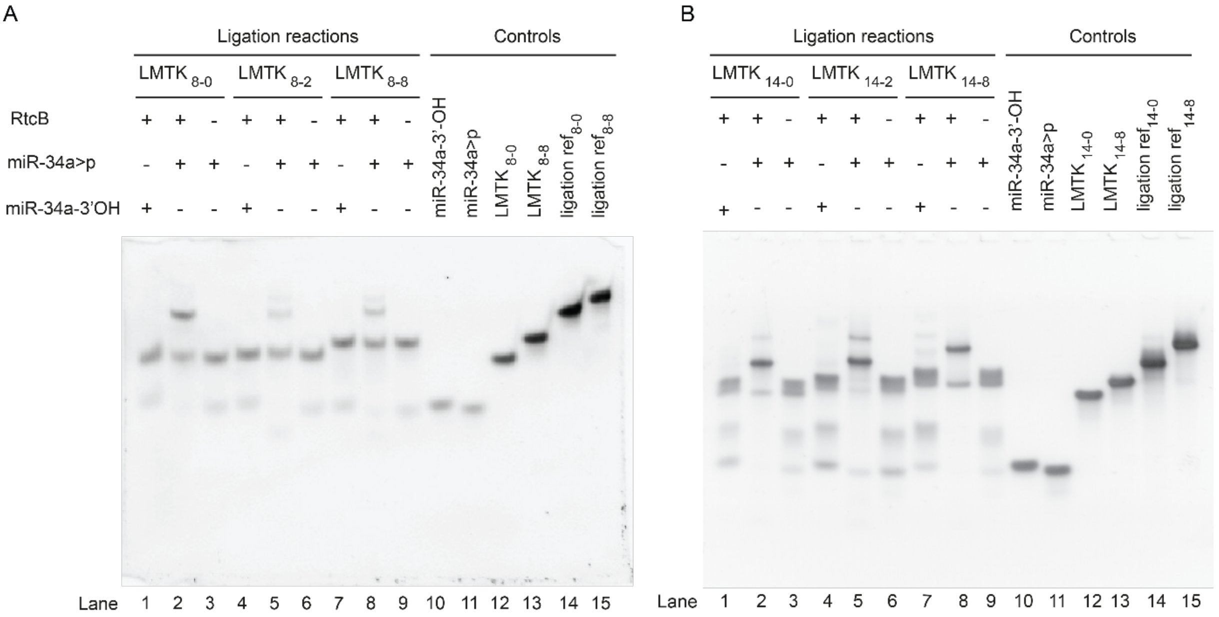

Figure S6. Full gel corresponding to **Fig. 4.** RtcB-mediated ligation of 2', 3'-cyclic phosphate miR-34a-5p strands to 5'-OH mRNA mimics. a) RtcB-mediated ligation of miR-34a-5p>p (22 nt) to different LMTK3 mRNA mimics (64-72 nt) with 8 consecutive and b) 14 consecutive complementary nucleotides and a variable 5' overhang of the LMTK3 counter-strands (0, 2 or 8 nt overhang). Reaction mixtures contained 0.5 µM RNA duplexes, 0.75 µM RtcB, 0.1 mM GTP, 1 mM $MnCl_2$ and 1x RtcB reaction buffer. Staining with SYBR Gold Nucleic Acid Gel Stain.

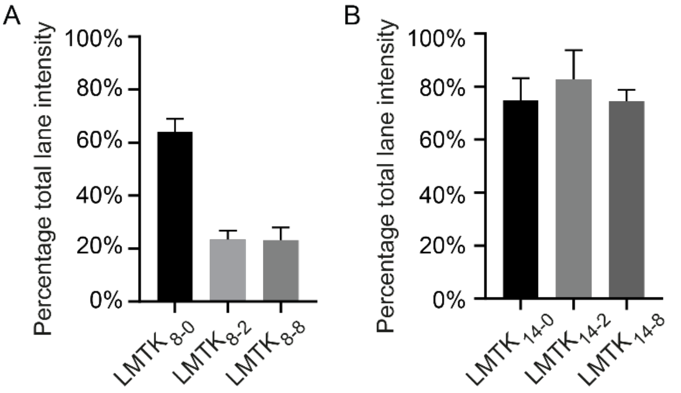

Figure S7. Ratio of intensity ligation product to total lane intensity from Fig. 4. The intensity ratio was used as a surrogate for ligation efficiency. A) Intensity ratios from Fig. 4a. B) Intensity ratios from Fig. 4b. Mean intensity ratio +/- SD shown from three independent replicates. Quantification using ImageJ.

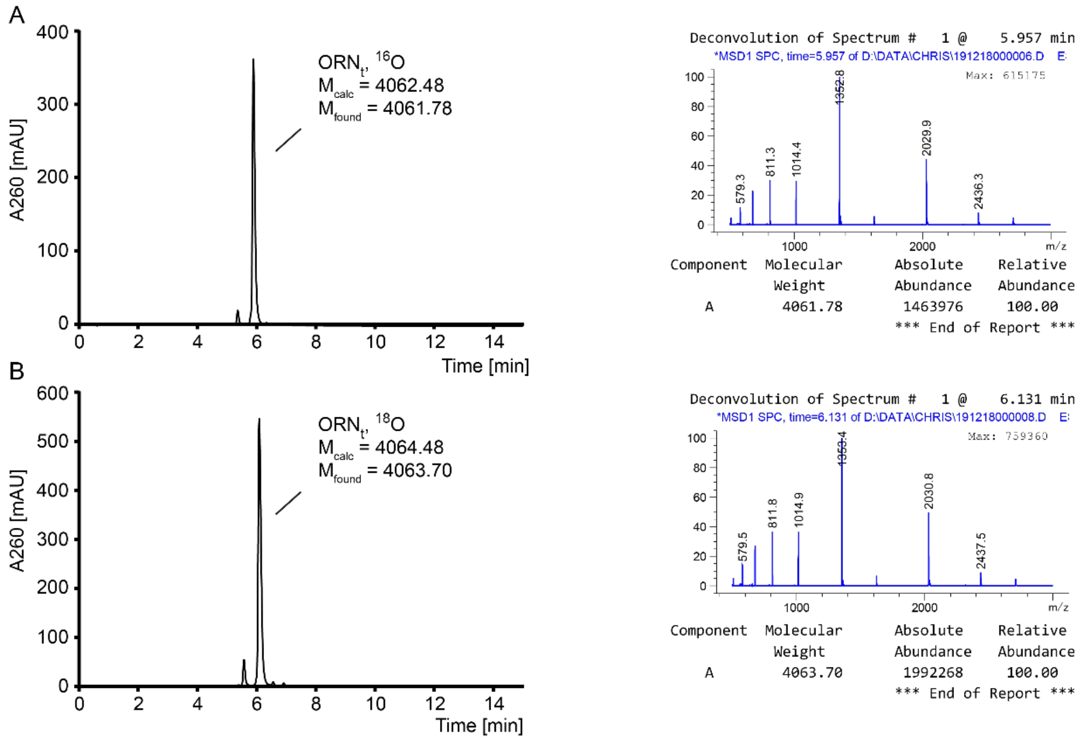

Figure S8. System suitability test to detect the difference between ^{16}O and ^{18}O containing oligonucleotides. A 13mer test RNA (ORN$_t$; 5'-AUGACUCUACUGU-3') containing either (A) all ^{16}O linkages or (B) a single ^{18}O-labelled 1inkage at the 3'-end. LC-MS analysis was performed on an Agilent 1200/6130 system fitted with a Waters acquity UPLC OST C-18 column (2.1×50 mm, 1.7 μm) at 65°C, with a gradient of 5-35% eluent B in 14 min with a flowrate of 0.3 mL min^{-1}. Eluent A was aqueous hexafluoroisopropanol (0.4 M) containing triethylamine (15 mM). Eluent B was methanol.

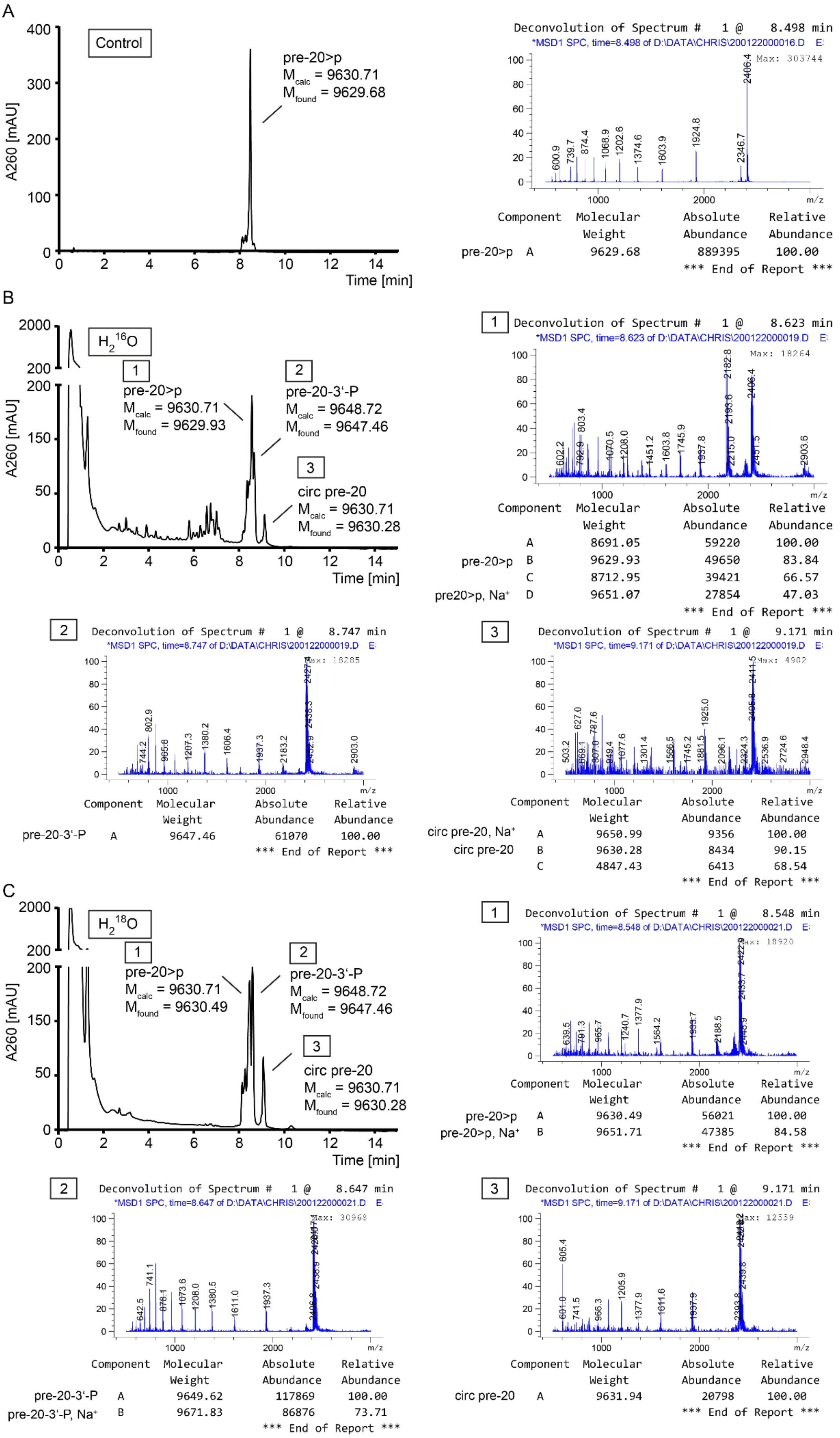
A
Control
pre-20>p
M_{calc} = 9630.71
M_{found} = 9629.68
A260 [mAU]
Time [min]
Deconvolution of Spectrum # 1 @ 8.498 min
*MSD1 SPC, time=8.498 of D:\DATA\CHRIS\200122000016.D E:
Max: 303744
Component Molecular Weight Absolute Abundance Relative Abundance
pre-20>p A 9629.68 889395 100.00
*** End of Report ***
B
$H_2{}^{16}O$
1
pre-20>p
M_{calc} = 9630.71
M_{found} = 9629.93
2
pre-20-3'-P
M_{calc} = 9648.72
M_{found} = 9647.46
3
circ pre-20
M_{calc} = 9630.71
M_{found} = 9630.28
A260 [mAU]
Time [min]
1 Deconvolution of Spectrum # 1 @ 8.623 min
*MSD1 SPC, time=8.623 of D:\DATA\CHRIS\200122000019.D E:
Max: 18264
Component Molecular Weight Absolute Abundance Relative Abundance
A 8691.05 59220 100.00
pre-20>p B 9629.93 49650 83.84
C 8712.95 39421 66.57
pre20>p, Na+ D 9651.07 27854 47.03
*** End of Report ***
2 Deconvolution of Spectrum # 1 @ 8.747 min
*MSD1 SPC, time=8.747 of D:\DATA\CHRIS\200122000019.D E:
Max: 18285
Component Molecular Weight Absolute Abundance Relative Abundance
pre-20-3'-P A 9647.46 61070 100.00
*** End of Report ***
3 Deconvolution of Spectrum # 1 @ 9.171 min
*MSD1 SPC, time=9.171 of D:\DATA\CHRIS\200122000019.D E:
Max: 4902
Component Molecular Weight Absolute Abundance Relative Abundance
circ pre-20, Na+ A 9650.99 9356 100.00
circ pre-20 B 9630.28 8434 90.15
C 4847.43 6413 68.54
*** End of Report ***
C
$H_2{}^{18}O$
1
pre-20>p
M_{calc} = 9630.71
M_{found} = 9630.49
2
pre-20-3'-P
M_{calc} = 9648.72
M_{found} = 9647.46
3
circ pre-20
M_{calc} = 9630.71
M_{found} = 9630.28
A260 [mAU]
Time [min]
1 Deconvolution of Spectrum # 1 @ 8.548 min
*MSD1 SPC, time=8.548 of D:\DATA\CHRIS\200122000021.D E:
Max: 18920
Component Molecular Weight Absolute Abundance Relative Abundance
pre-20>p A 9630.49 56021 100.00
pre-20>p, Na+ B 9651.71 47385 84.58
*** End of Report ***
2 Deconvolution of Spectrum # 1 @ 8.647 min
*MSD1 SPC, time=8.647 of D:\DATA\CHRIS\200122000021.D E:
Max: 30968
Component Molecular Weight Absolute Abundance Relative Abundance
pre-20-3'-P A 9649.62 117869 100.00
pre-20-3'-P, Na+ B 9671.83 86876 73.71
*** End of Report ***
3 Deconvolution of Spectrum # 1 @ 9.171 min
*MSD1 SPC, time=9.171 of D:\DATA\CHRIS\200122000021.D E:
Max: 12339
Component Molecular Weight Absolute Abundance Relative Abundance
circ pre-20 A 9631.94 20798 100.00
*** End of Report ***

Figure S9. Full chromatograms and mass spectrometry from Fig. 3. A) Synthetic pre-20>p alone. B) RtcB mediated ligation of pre-20>p in $H_2{}^{16}O$ and (C) $H_2{}^{18}O$. Signal 1 corresponds to unligated (linear) pre-20>p (M_{calc} = 9630.71). In both, $H_2{}^{16}O$ and $H_2{}^{18}O$ also the Na^+ adduct was detected. In the ligation under $H_2{}^{16}O$ conditions, components A and C (signal 1) could not be assigned and are likely to be impurities. Signal 2 corresponds to the RNA-3'-phosphate intermediate (M_{calc} = 9648.72 with ^{16}O and 9650.72 with ^{18}O, respectively). Signal 3 corresponds to circular pre-20>p after ligation (M_{calc} = 9648.72 with ^{16}O and 9650.72 with ^{18}O, respectively). Deconvoluted ion sets for the calculation of the respective components corresponding to pre-20>p, pre-20-3'-P and circ pre-20 under each signal are shown in Figure S 8. RtcB ligation was performed at 37°C for 1 h in the presence of 50 mM Tris.HCl (pH = 8), 1 mM $MnCl_2$, 0.1 mM GTP, 0.75 µM RtcB and 0.5 µM pre-20>p. Ligation was quenched through the addition of Na_2EDTA to a final concentration of 50 mM. LC-MS analysis was performed as described in Fig. 5 and Fig. S 2.

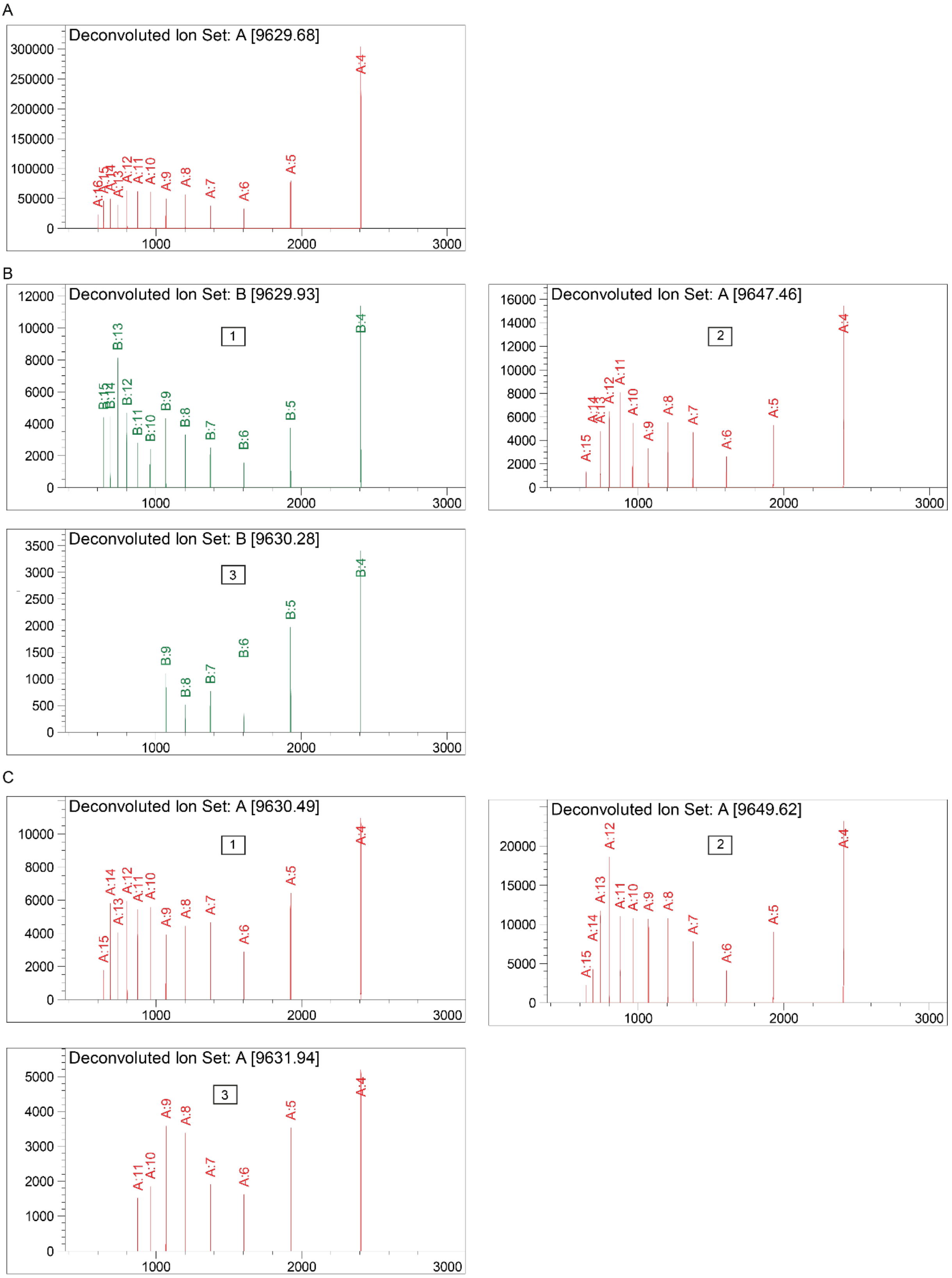

Figure S10. Deconvoluted ion sets from Figure S9. A) Deconvoluted ion set for the calculation of component A (pre-20>p) in Fig. S 7A. B) Deconvoluted ion sets for the calculation of signal 1, component B (pre-20>p); signal 2, component A (pre-20-3'-P); signal 3, component B (circ pre-20) in Fig. S 7B. C) Deconvoluted ion sets for the calculation of signal 1, component A (pre-20>p); signal 2, component A (pre-20-3'-P); signal 3, component A (circ pre-20) in Fig. S 7C.

Table S1. Sequences of oligoribonucleotides used in this study.

oligonucleotide	Sequence (5'-3')
miR-34a-5p	UGGCAGUGUCUUAGCUGGUUGU (>p)
miR-34a-3-p	CAAUCAGCAAGUAUACUGCCCU
miR-106a-5p	AAAAGUGCUUACAGUGCAGGUAG (>p)
miR-106a-3p	CUGCAAUGUAAGCACUUCUUAC
Let-7g-5p	UGAGGUAGUAGUUUGUACAGUU (>p)
Let-7g-3p	CUGUACAGGCCACUGCCUUGC
ORN_t	AUGACUCUACUGU
Pre-20>p	UGCAGGUAGUUUUGGCAUGACUCUACUGUA>p
ORN_{64}>p	UCAAGAGACUAAAUCCUUCCAUGAAUAGUUUUCAAGAGAAAACUAUUCAUGGAAGGAUUUAGU>p
$LMTK_{8-0}$	GUGGAUGACGGCGCCACUGCCACCACCGCAGACGCCGCCUCU
$LMTK_{8-2}$	CUGUGGAUGACGGCGCCACUGCCACCACCGCAGACGCCGCCUCU
$LMTK_{8-8}$	AAUCCUCUGUGGAUGACGGCGCCACUGCCACCACCGCAGACGCCGCCUCU
Ligation $product_{8-0}$	UGGCAGUGUCUUAGCUGGUUGUGUGGAUGACGGCGCCACUGCCACCACCGCAGACGCCGCCUCU
Ligation $product_{8-8}$	UGGCAGUGUCUUAGCUGGUUGUAAUCCUCUGUGGAUGACGGCGCCACUGCCACCACCGCAGACGCCGCCUCU
$LMTK_{14-0}$	GUGGAUGACUAAGACACUGCCACCACCGCAGACGCCGCCUCU
$LMTK_{14-2}$	CUGUGGAUGACUAAGACACUGCCACCACCGCAGACGCCGCCUCU
$LMTK_{14-8}$	AAUCCUCUGUGGAUGACUAAGACACUGCCACCACCGCAGACGCCGCCUCU
Ligation $product_{14-0}$	UGGCAGUGUCUUAGCUGGUUGUGUGGAUGACUAAGACACUGCCACCACCGCAGACGCCGCCUCU
Ligation $product_{14-8}$	UGGCAGUGUCUUAGCUGGUUGUAAUCCUCUGUGGAUGACUAAGACACUGCCACCACCGCAGACGCCGCCUCU
ORN_b	CCACAGAGGAUUACAACCAGCUAA
siRenilla	AS: UUUCUCGCCCUCUUCGCUCUU<br>SS: GAGCGAAGAGGGCGAGAAAUU
siCon	AS: GUGNNUAANNAACNCANACTT<br>SS: GUNUGNGUUNNUUANNCACTT

The presence of a 2', 3'-cyclic phosphate terminus is denoted as ">p". In all other cases, RNAs are terminated with a 3'-OH group. N= randomized position, AS= antisense strand, SS= sense strand.

Table S2. Sequences of DNA inserts for Dual Luciferase reporter plasmids

**Reporter plasmids**	**Sequence (5'-3')**
hsa-miR-106a (1x target site, fully complementary)	*CCTCCACTTCAGCCAGGA*CTCGAGGTTAGGGGTATATGATGGGGGAGTAGATCTTTCTAGGAGGGAGACACTGGCCCCTCAAATCGTCCAGCGACCTTCCTCATCCACCCCATCTACCTGCACTGTAAGCACTTTTGATTAGCAGCGGAACAAGGAGTCAGACATTTTAAGATGGCGGCCGC*TGAGTCTTCGGACCTCGC*
hsa-miR-34a (3x target sites, fully complementary)	*CCTCCACTTCAGCCAGGA*CTCGAGAGGGTTTGAAATATAGCTGTTCTTTATGCATAAAACACACAACCAGCTAAGACACTGCCAGAGAAAAAAATCGTATTGAATGGCCATTTCCCTACTTATAAGATGTCTCAATCTGAATTTGCGGCCGC*TGAGTCTTCGGACCTCGC*
hsa-let-7g (1x target site, fully complementary)	*CCTCCACTTCAGCCAGGA*CTCGAGTTTTCATTTTGAAGGGGCCTCACCGAGTGGGGGCATCATCAAAAACTTTAACTATACAACCTACTACCTCAGGAGTCCCCTCACCTCCTCTAAGGTTGGGCAGGGTGACCCTGAAGTGAGCACAGCCTAGGGCTGAGCTGGGGACCTGGTGCGGCCGC*TGAGTCTTCGGACCTCGC*

Restriction sites are underlined, primer binding sites are indicated as italicized letters.

REFERENCES

1. Röthlisberger, P., Berk, C. & Hall, J. (2019) RNA Chemistry for RNA Biology, *CHIMIA International Journal for Chemistry*. **73**, 368-373.
2. Laikhter, A., Srivastava, S. C. & Srivastava, N. (2013) Synthesis of 2', 3'—and 3', 5'—cyclic phosphate mono-and oligonucleotides in, Google Patents,
